# A Focus Group Study Among Inactive Adults Regarding the Perceptions of a Theory-Based Physical Activity App

**DOI:** 10.3389/fpubh.2021.528388

**Published:** 2021-06-18

**Authors:** Nicky Nibbeling, Monique Simons, Karlijn Sporrel, Marije Deutekom

**Affiliations:** ^1^Centre of Expertise Urban Vitality, Amsterdam University of Applied Sciences, Amsterdam, Netherlands; ^2^Chair Group Consumption and Healthy Lifestyles, Wageningen University & Research, Wageningen, Netherlands; ^3^Department of Human Geography and Planning, Utrecht University, Utrecht, Netherlands; ^4^Faculty of Health, Sports and Social Work, Inholland University of Applied Sciences, Haarlem, Netherlands

**Keywords:** focus group, physical activity, persuasive strategies, mobile application, behavior change

## Abstract

**Background:** Despite the increasing attention for the positive effects of physical activity (PA), nearly half of the Dutch citizens do not meet the national PA guidelines. A promising method for increasing PA are mobile exercise applications (apps), especially if they are embedded with theoretically supported persuasive strategies (e.g., goal setting and feedback) that align with the needs and wishes of the user. In addition, it is argued that the operationalization of the persuasive strategies could increase the effectiveness of the app, such as the actual content or visualization of feedback. Although much research has been done to examine the preferences for persuasive strategies, little is known about the needs, wishes, and preferences for the design and operationalization of persuasive strategies.

**Objective:** The purpose of this study was to get insight in the needs, wishes, and preferences regarding the practical operationalization of persuasive strategies in a mobile application aimed at promoting PA in healthy inactive adults.

**Methods:** Five semistructured focus groups were performed. During the focus groups, the participants were led into a discussion about the design and operationalization of six predefined theory-based persuasive strategies (e.g., self-monitoring, feedback, goal setting, reminders, rewards, and social support) directed by two moderators. The audio-recorded focus groups were transcribed verbatim and analyzed following the framework approach.

**Results:** Eight men and 17 women between 35 and 55 years (mean age, 49.2) participated in the study. Outcomes demonstrated diverse preferences for implementation types and design characteristics of persuasive strategies in mobile applications. Basic statistics (such as distance, time and calories), positive feedback based on easy-to-achieve goals that relate to health guidelines, and motivating reminders on a relevant moment were preferred. Participants had mixed preferences regarding rewards and a social platform to invite other users to join PA.

**Conclusions:** Findings indicated that in mHealth applications for healthy but inactive adults, persuasive strategies should be designed and implemented in a way that they relate to health guidelines. Moreover, there is a need for an app that can be adapted or can learn based on personal preferences as, for example, preferences with regard to timing of feedback and reminders differed between people.

## Introduction

Over the past decades physical inactivity has negatively impacted the health of Western populations (1). Together with unhealthy diets, physical inactivity represents the major risk factor for various non-communicable diseases, including cancer, cardiovascular disease, and type 2 diabetes (1, 2). In recognition of the strong relation between physical inactivity and non-communicable diseases, the World Health Organization (WHO) has set the objective to reduce the prevalence of insufficient physical activity (PA) by 10% by 2025 (3). Despite of multiple efforts worldwide that aim to enhance PA, a meta-analysis including 1.9 million participants demonstrated that the prevalence of people who do not meet the recommended level of PA (42.3% in high-income Western countries) remains constant in the 15 years between 2001 and 2016 (4). If this trend continues, the global PA target of 2025 will not be reached.

In the Netherlands, similar developments can be observed with regard to PA. In 2017, only 49% of Dutch adults met the Dutch PA guideline of 150-min moderately intensive exercise a week and strength exercises two times a week. Adults and elderly are the groups that perform worst concerning these guidelines (5). In answer to these developments, advocating an active lifestyle is recognized as an immediate public health priority by the WHO (6). This has encouraged governments and public institutions to initiate a multitude of interventions that aim to induce behavior change and enhance PA.

Among the developments are electronic health (eHealth) and mobile health (mHealth) technologies, as, for example, smartphone applications (apps), that show promising results to stimulate PA (7–9). Exercise apps can be utilized as cost-effective interventions that can reach large populations (10), especially if they are embedded with theoretically supported persuasive strategies that align with the needs and wishes of the user.

However, despite the emerging field of exercise apps, and evidence on short-term effectiveness, little is known on their effectiveness on the long term. More specifically, which functionalities in these app based interventions are effective and which are not is still an open question (11). In addition, user engagement appears a challenge, especially on the long term (8). In eHealth interventions, user engagement usually declines after the first few weeks as people often lack commitment to use the app (12). It is important that people keep using the app, as app engagement has been linked to sustained PA behavior change (13). Similar, there appears to be little clarity on the most effective way to implement these strategies into mobile exercise apps for different target groups (8).

To increase user engagement with exercise apps, more knowledge is needed about what app features and behavior change techniques (BCTs) people favor and use (14). Persuasive strategies, also called behavior change techniques (14), are elements of interventions that attempt to influence people's behavior, such as rewards or reminders (15). These elements are often grounded in behavioral theories, such as the transtheoretical model, the theory of reasoned action, and the theory of planned behavior (16). Persuasive strategies play an important role in applications that aim for behavior change (17), and interventions that incorporate behavior change techniques have been associated with greater effectiveness (18). Recently, a scoping literature review by Sporrel et al. identified the following six strategies as most promising to increase PA in mobile exercise interventions: feedback, self-monitoring of behavior, goal setting, reminders, rewards, and social influence (19).

These six strategies are, however, still quite generic. For example, research shows that self-monitoring is a promising strategy to implement in an exercise app to increase PA (7). However, it remains unclear what people would prefer the app to monitor exactly: their speed, distance covered, or, for example, their calories burned. In other words, research does not explain how the strategies should be used in practice, that is, when, where, in which shape, and for whom they will be effective (14).

To answer this question, it is important to gain insight into the needs, wishes, and preferences of end-users of exercise apps. Involving potential end users in the design of technologies such as apps has previously been found to positively affect usability and user satisfaction (20, 21). Consequently, involving end users resulted in higher acceptance and more effective use of these technologies (21). Therefore, the aim of the current study is to gain insight in the perspectives regarding the practical operationalization of persuasive strategies in a mobile application aimed at promoting PA in healthy inactive adults.

## Method

### Participants

Adult urban residents living close to city parks in Amsterdam were invited to participate. Participants were recruited through community centers located in Amsterdam and through Facebook campaigns. The following criteria had to be met for inclusion: (1) in possession of a smartphone, (2) native Dutch speaker, (3) self-indicated ableness to exercise (walking and running), (4) not meeting the Dutch exercise guidelines of 150 min a week moderate intense exercise and two times strength exercises [self-reported; (22)], and (5) being in the contemplation or preparation phase according to the transtheoretical model of behavior change (23). People in these two phases were targeted because this group is aware and willing to change their behavior. They can be defined as finding it hard to initiate and maintain PA on a regular basis (24). People in the precontemplation phase were excluded, as they do not have the intention to change and are therefore unlikely to download an exercise app. State of change with regard to physical activity was measured by the flow chart of Ronda et al. (25).

### PAUL Project

This study is part of the larger Playful data-driven Active Urban Living (PAUL) project, which was initiated in 2017. The goal of PAUL is to motivate Dutch inactive urban residents to increase their PA through walking, running, and strength exercises in urban spaces (such as parks) with the use of a theoretically and empirically driven app: the PAUL app. The development of the PAUL app arises from interdisciplinary research from the fields of psychology, movement sciences, computer science, and artificial intelligence (26). Three pillars are at the basis of the development of the PAUL app. The first pillar is data-driven research. Data from existing exercise apps were analyzed to identify which situations are associated with running behavior (frequency and duration) of infrequent app users (27, 28). The results of this data mining study was applied to determine the timing of the reminders, which will be further personalized during app use by a reinforcement learning model.

The second pillar comprises a literature study that was performed to identify the most promising behavior change techniques, also called persuasive strategies, to incorporate in the app to enhance PA. Sporrel et al. (19) identified the following six strategies as most promising to increase PA in mobile exercise interventions: feedback, self-monitoring of behavior, goal setting, reminders, rewards, and social influence (see Table 1).

**Table 1:** Most promising persuasive strategies according to Sporrel et al. (19) with their definitions.

**Persuasive strategy**	**Definition**
Feedback	System provides the participant with feedback about their own recorded behavior, commenting on a person's behavioral performance, or a discrepancy between one's own performance in relation to others.
Self-monitoring	System should provide means for users to track their performance or status.
Goal setting	The system encourages to make a behavioral resolution (e.g., engage in more exercise next week). This is directed toward encouraging people to decide to change or maintain change.
Reminders	System should remind users of their target behavior while using the system.
Reward	System should provide virtual rewards for users to give credit for performing target behavior.
Social influence	System should provide means for comparing performance with the performance of others.

The third pillar comprises the current study, in which the potential end user is involved in the design of the app. Preferences regarding implementation types and design characteristics of six persuasive strategies (feedback, self-monitoring of behavior, goal setting, reminders, rewards, and social influence) were explored using a qualitative research design (focus groups).

### Study Design and Procedure

Focus groups were conducted, as this approach is suitable to provide insight in the underlying assumptions that gave rise to the views and opinions of people, such as the potential end users of an app (29). Moreover, it stimulates interaction between potential users with the hopes of obtaining additional insight into the topic of interest (30). Five focus groups with five to eight participants were performed in the spring of 2019. We limited the number of participants for each focus group to a maximum of eight participants to facilitate a good discussion, in which all participants have the opportunity to contribute (31). Ethical approval for the study was obtained from the ethics review board of the Utrecht University.

As in qualitative research, sample size cannot be determined; focus groups were held until saturation of new information was reached (32). Moreover, to make sure no new information was missed, one additional focus group session was organized after the estimated theoretical saturation. Data of this additional focus group session were included in the results. Focus group sessions were conducted in a private room at the Amsterdam University of Applied Sciences (HvA) and VU University. The sessions consisted of two parts of 45 min with a break of 10 min in between. All five sessions were audio and video recorded and facilitated by two researchers. One researcher guided the discussion, and the assisting researcher took notes.

On arrival, all participants gave written informed consent and completed a short questionnaire on age, gender, ethnicity, app use, and self-reported skill in app use. Next, a short introduction on the PAUL project was provided, and examples of currently available exercise applications and their content were outlined. A digital presentation (Powerpoint slides) was used to guide the group through the different topics. To make participants feel at ease, the discussion started with a warming-up round in which participants introduced themselves and shared their favorite app.

Next, the six persuasive strategies were discussed. A semistructured topic list with the six predetermined persuasive strategies and corresponding open questions guided the discussion. The six strategies were discussed in the following order: monitoring, feedback, and goal setting before the break; then, reminders, social influence, and rewards after the break. Besides the digital presentation, the researchers included short questionnaires on preference for rewards and optimal time of the day to receive reminders for exercise through the app. After filling in the questionnaire, the questions were also discussed by the groups. As a reward, the participants received a gift voucher of €20 for participating in the study.

### Topic List

The topic list was structured according to the six (theory-based) selected persuasive strategies (Table 2). For each of the strategies, subtopics regarding the operationalizations of the strategies were drafted by a team of researchers from different disciplines. The subtopics were based on findings from earlier literature reviews on promising operationalizations of persuasive strategies (19, 33), behavior theories, and findings of the empirical data mining study (28).

**Table 2:** Topic list of the focus group.

**Interview topics**	**Questions**
1. Self-Monitoring	a. How do you feel about monitoring your PA during walking or running?
	b. What kind of statistics would you like to see in an exercise app?
2. Feedback	a. How do you feel about receiving feedback on your exercise behavior through an app?
	b. How would you like to receive feedback through the app?
	c. What type of feedback would you like to receive?
3. Goal setting	a. How do you feel about setting your own goals in an exercise app?
	b. What kind of goals would you like to set?
	c. How and to what extent would you like the app to provide suggestions for goal setting (e.g., choice between easy and more difficult goals)?
4. Reminders	a. How do you feel about receiving reminders to exercise through an app?
	b. What kind of reminders would work best for you?
	c. At what moment(s) would you prefer to receive the reminder?
5. Social influence	a. How could an exercise app provide for social support?
	b. Would you like to be able to share your results with others?
	c. How do you stand against competitive elements in an exercise app?
6. Rewards	a. How and to what extent would you like the app to reward you for reaching your PA targets?
	b. When would you like to receive rewards (e.g., related to gain in fitness)?

### Data Analysis

The video-recorded data of the focus groups were transcribed verbatim. The data were coded and analyzed with ATLAS.ti version 8.0 (ATLAS.ti Scientific Software Development GmbH, Berlin, Germany). During coding, participants' names were pseudonymized. In order to structure the raw data into codes and themes, the five steps, namely, (1) familiarization, (2) identifying a thematic framework, (3) indexing, (4) charting, (5) mapping and interpretation, of the “framework approach” as presented by Pope et al. (34) were followed. This approach is specifically developed for studies that combine deductive and inductive methods (34). The coding started deductively using the persuasive strategies derived from behavioral change theories as point of departure. Subsequently, in a more inductive way, issues and experiences that raised from the discussion around these persuasive strategies (and other issues around exercise apps) were employed.

In step 1 of the framework approach, researchers got familiarized with the data by reading the transcripts. In step 2, a thematic framework was followed based on the theory-based persuasive strategies. Next, additional codes and themes were derived from the data. In step 3 and 4, codes were assigned to quotations, and then, these codes were rearranged to the right part of the thematic framework. Finally, the analyzing process was completed with an interpretation and the mapping of the data. The coding of the data was conducted by two researchers (KC^1^ and NN) independently. Findings were compared, and differences were discussed until consensus was reached.

## Results

### Participants

Twenty-five participants participated in the focus groups, of which 17 were female and 8 were male. The characteristics of the participants are provided in Table 3.

**Table 3:** The characteristics of the 25 participants.

**Characteristics**		***n* (%)**
**Mean age (SD) years**		49.2 (9.2)
**Gender**		
	Male	8 (32)
	Female	17 (68)
**Origin**		
	Dutch	18 (72)
	Western immigrant	2 (8)
	Non-western immigrant	5 (20)
**Education level**		
	Primary school	2 (8)
	Secondary school	0 (0)
	High school	7 (28)
	Bachelor's degree	10 (40)
	Master's degree or higher	6 (24)
**Smartphone use per day**		
	<1 h	1 (4)
	1–2 h	12 (48)
	2–3 h	3 (12)
	3 + h	9 (36)
**Self-reported skill with new smartphone applications**		
	Not very skillful	0 (0)
	Somewhat skillful	6 (24)
	Very skillful	19 (76)
**Interested in new smartphone applications for physical activity**		
	Not very interested	0 (0)
	Somewhat interested	7 (28)
	Very interested	18 (72)
**Application use for physical activity**		
	Yes	16 (64)
	No	9 (36)
**Phase of behavior change**		
	Preparation	5 (20)
	Contemplation	20 (80)

### Perceptions Regarding Persuasive Strategies

The results are presented in six sections that correspond to the six persuasive strategies: (1) self-monitoring, (2) feedback, (3) goal setting, (4) reminders, (5) social influence, and (6) rewards. Illustrative quotations are outlined per strategy in Tables 4, 5 divided per subtopic.

**Table 4:** Illustrative quotations self-monitoring, feedback, and goal setting.

**Topic**	**Subtopic**		**Illustrative quotations**
Monitoring	Health guidelines	Q1	“Yes, exactly, how much do we need to exercise to stay fit? What is the standard, what is general energy use?” [male, 35, preparation phase]
	Measuring units	Q2	“No, it is more, purely out of interest from how active I am in a day. Hours or minutes a day, I like to know that.” [female, 44, preparation phase]
	Presentation statistics	Q3	“I also like simple. Preferably also graphically, I always find figures better than numbers.” [male, 51, contemplation phase]
	Manually add exercise	Q4	“I don't want to be reached at any hour, you know. Then it would be nice to add your walk later.” [female, 39, preparation phase]
	Route map	Q5	“that you can say, well, next day, I want the same route but a bit longer or a bit different… and you can see how far you got in a certain area.” [female, 50, preparation phase]
Feedback	Framing	Q6	“And eh, no, I thought it would be nice if she gave you a pat on the back. And negative is not really eh, conducive.” [male, 47, preparation phase]
	Related to goal	Q7	“And what is also nice is if you, if you set a distance or a goal that the app after expiration, if you stop the app that indicates that: you have ‘so much%’ of your goal Or ‘so many miles to go”’ [female, 39, preparation phase]
	Turn on/off	Q8	“At a certain point it was annoying. [The last app]…gave feedback after 1 km already or something.” [female, 53, contemplation phase]
	Mode of delivery	Q9	“Very short, so you don't have to stop and lose your pace. Just really short, powerful.” [female, 39, preparation phase]
	Timing of feedback	Q10	“I'm just thinking during, actually. Then I can change something.” [male, 35, preparation phase]
Goal setting	Appreciation goal setting	Q11	“Yes, you have to set goals. Because otherwise you won't make it.” [female, 55 preparation phase]
	Health guidelines	Q12	“Well, the curve of how much exercise should you do? I would like that. I will come back to that but that is really nice. Because a lot of things are being said that you have to sit less, it is unhealthy, you have to move more. Yes, I still wonder, when are you moving enough?” [female, 44, preparation phase]
	Sub goals	Q13	“Yes, so you have a larger goal, an end goal, that 10K, maar divided into small steps. So you can achieve it. That is important to stay positive.” [male, 51, preparation phase]
	Appropriateness to current abilities	Q14	“Yes, an example of a goal can be: Try to walk for 10 consecutive minutes today. I think that is a little more realistic if you want to motivate people. That people then say: I can do that…Instead of immediately want to take a million steps.” [female, 39, preparation phase]
	Suggestions for goals	Q15	“Yes, yes depends on it, because, yes, I think, it must be possible to enter data in the app. About weight, height etc. that data depends. And if that says, this is a good fit for you. That would be nice. But not just, generally, for everyone.” [male, 35, preparation phase]
	Measuring units	Q16	“I would like that, a kind of fitness app. And then your goal is strengthening your muscles and you can do exercises every day.” [female, 53, contemplation phase]

**Table 5:** Illustrative quotations reminders, social influence, and rewards.

**Topic**	**Subtopic**		**Illustrative quotations**
Reminders	Appreciation reminders	Q17	“Yes, but then I could set, for example, I name something, on that and that day, I don't want a reminder. No reminder as standard on that and that day.” [female, 51, preparation phase]
	Content	Q18	“They [reminders] must therefore be personal and realistic in a friendly way. They should be adjusted to my own goal so they are relevant.” [female, 52, preparation phase]
	Time of day	Q19	“Because it is inconvenient if you receive such a message when you are in a meeting” [female, 52, preparation phase]
Social influence	Safety	Q20	“But then people have to be honest, cause you also have crazy people, that is a bit scary then. Especially if you go running in the evening.” [female, 53, contemplation phase]
	Sharing results	Q21	“For me it is actually, yes, I do not want the other person to know that I exercise so little.” [female, 50, preparation phase]
	Privacy	Q22	“Yes, privacy, what are they going to do with your data?” [male, 35, contemplation phase]
	Competition	Q23	“I would like a kind of virtual opponent. If I just run my virtual time, if I run better than my average, it indicates that.” [female, 47, preparation phase]
Rewards	Appreciation rewards	Q24	“I like rewards. I mean, at the end, you feel proud that you made it. And I achieved those goals. And then I want to do more for my health.” [female, 54, preparation phase]
	Terms for reward	Q25	“Yes, in phases as a kind of stimulus. Start easy and then it becomes harder [to receive a reward].” [female, 53, contemplation phase]
	Compliments	Q26	“Well I think such a cup is a bit childish. Just like in the old days, such a teacher sticker in the classroom or something. I like it when you do something, more feedback like; you are on track or you are very well on your way to your goal or something.” [female, 47, preparation phase]
	Trophies	Q27	“Such a trophy is fun.” [female, 39, preparation phase]
	Physical rewards	Q28	“Yes, well I already have eh, also written down for example or a discount at a gym or eh.” [female, 50, preparation phase]

#### Self-Monitoring

With regard to the monitoring of their PA with an exercise app, five subtopics could be distinguished from the participants quotes (health guidelines, measuring units, presentation statistics, manually add exercise, and route map; see Table 4). The main reason for participants to use an exercise app would be that they can monitor their health status. They preferred incorporation of the physical activity guidelines in the app, so they would be able see when their PA is sufficient for a healthy lifestyle (Q1). Thereby, participants would like to track their physical activity during the entire day to be able monitor their progress toward such a health goal (Q2). However, the kind of statistics favored by the participants differed very much. Most popular measuring units for statistics were exercise time, distance, and calories burnt. In addition, number of steps, heartbeat, speed, and height traveled were mentioned. Preferably, statistics are presented in graphs and tables rather than numbers, as that provides a quick, easy, and visually attractive oversight of their statistics (Q3). Participants suggested to include an option in the app that enables them to manually add physical activities. This is considered a valuable addition, as participants do not always carry their smartphone with them, yet they want to make sure that all PA they performed that day is included (Q4). In addition, a map showing the walked route was considered useful, as it enables them to collect attractive routes that they can reuse, adjust, or extent (Q5).

#### Feedback

Five subtopics on how participants want to receive feedback from the app emerged from the focus group conversations (framing, turn on/off, content, mode of delivery, and timing of feedback; see Table 4). Participants suggested that the app should provide positive and motivating feedback. This type of feedback was perceived as favorable, as it would boost their motivation and encourage them to persist, while negative feedback, on the other hand, would demotivate them (Q6). The majority of the participants suggested that feedback should be tailored to their exercise goals and especially to their progress with regard to reaching these goals (Q7). However, some participants found feedback they experienced with applications in the past annoying and stressed out that they would want to be able to turn the feedback on or off (Q8). The mode of delivery and timing of the feedback were also points of concern. Participants explained that looking at their phone during PA was not practical. Therefore, they prefer to receive very short feedback, and preferably audio, such as a sound (a “pling”) half way through their exercise time or a spoken message (Q9). With regard to timing of feedback (before„ or after exercise), personal preferences were considerably divided. Some indicated that they do not want to receive feedback during the session but only after because then it would contain the most information and a full overview of their performance. On the other hand, another participant mentioned he only wanted feedback during the physical activity as only then he could still act upon the feedback (Q10).

#### Goal Setting

For a future exercise app, participants' quotes revealed six subtopics with regard to how goal setting should be represented in the app (appreciation goal setting, health guidelines, subgoals, appropriateness to current abilities, suggestions for goals, and measuring units; see Table 4). Overall, goal setting was deemed positive by the participants, as by many, it was considered necessary to achieve their targets (Q11). In line with the previous topics, preferences for goal setting were mostly related to participants' need for an exercise app to help them to achieve PA guidelines to stay healthy (Q12). Participants indicated that when end goals lie in the distant future, an app would be helpful to be able to set short-term subgoals. They argued that this would make the end goal more accessible, which in turn would help them to stay positive in pursuing the end goal (Q13). Participants prefer goals that are appropriate to their current abilities. Goals that are in line with their abilities make it easier to start exercising, as they are rewarded for their effort sooner and the success experience of reaching this goal motivates them to pursue the next sub goal (Q14). Nevertheless, participants find it hard to set their own goals. For example, they manage to set an end goal but do not know how to build up to that goal. Therefore, they would prefer an app to provide suggestions for goals, tailored to the user (Q15). Those goals are preferably set in terms of exercise time and distance. In addition, health-related goals, such as calories and losing weight, were brought up. Participants expressed a wish for the possibility to set a goal to perform muscle strengthening exercises because this would be a novel functionality that is not yet included in commercial apps (Q16).

#### Reminders

The questions whether and how reminders should be provided by the app revealed three subtopics (appreciation of reminders, time of day, and content; see Table 5). Reminders were appreciated by most of the participants, as they were perceived as motivating and they can function as a lever to perform PA. However, some perceived reminders as annoying and were stringent on the wish not to receive them. In reaction to this discussion, participants suggested to let the user choose by him- or herself to turn reminders on or off in different occasions (Q17). The content of the reminders should be friendly and fun because that was perceived as most motivating. Moreover, participants preferred reminders that are personalized, for example, in line with someone's goal (Q18). They argue that it is more motivating when the message is specifically directed toward them, instead of having the feeling that they get an automatically generated message that is the same for everyone. There was no consensus between the participants on the timing of reminders. Answers varied from early in the morning till late in the evening and from 2 h to 15 min before an exercise activity and during working hours or, exactly the opposite, preferably not during working hours, as it would disturb them during work or meetings (Q19).

#### Social Support

With regard to the conversation about possible social influences of an exercise app, four subtopics emerged (framing, turn on/off, content, mode of delivery, and timing of feedback; see Table 5). Different options for offline (in the app) as well as online (in real life) social support were brought up. Only a minority of the participants actually wanted to meet new people through the application. Uncertainty about safety of such a meeting was the major argument. Especially, female participants argued that they would not want to meet a man through the app because they would feel unsafe (Q20). As a solution, participants suggested to create a platform inside the application, such as a bulletin board, where you can ask for a companion to join physical activities. Notably, the majority of participants did not feel the need to share results with others on social media or on an internal platform of the app. The most mentioned reasons for this were that they were not “proud” of their physical activity results or that they did not find it important enough to share it with others (Q21). However, an internal platform was preferred over sharing on social media because participants were reluctant to share data with a wide audience for privacy reasons (Q22). Participants also explained that competition with others does not motivate them to engage in PA. That is, almost none of the participants wish to beat others in, for example, a competitive exercise game. The main reason for this was that the participants' feeling was that they would lose anyway. However, several participants indicated that competition could be fun or motivating if you could compete against your virtual self because then your personal growth would be rewarded (Q23).

#### Rewards

The conversation on whether an exercise app should provide rewards resulted in five subtopics (appreciation rewards, terms for reward, compliments, trophies, and physical rewards; see Table 5). Most participants indicated that they preferred an app that gives rewards after achieving a goal because they believe this would make them feel proud, stimulates them to use the app more, and consequently perform more PA (Q24). Moreover, the terms on which you receive a reward preferably builds up with difficulty level because then it provides a stimulus to increase their effort (Q25). Preference for the type of reward varied widely between participants. For some participants, a compliment or a “thumbs-up” is already motivating (Q26). Others indicated that they would like a digital reward in the form of a trophy, medal, or batch (Q27). Others found such a type of reward childish and would prefer a reward in real life such as discounts on (healthy) products, or they would like to collect points such as air miles (Q28).

## Discussion

In the current study, we explored the needs and wishes of inactive Dutch urban residents regarding the design characteristics and technical implementations of six promising persuasive strategies (monitoring, feedback, reminders, goal setting, social support, and rewards). The results indicate that inactive urban residents prefer basic, health-related feedback linked with ability-appropriate goals and positive reminder messages to encourage them to be more physically active. Moreover, preferences differ between people, showing a need for an app that is personalized and can be adapted based on these personal preferences.

Thus far, little is known about how to make the translation from theoretically embedded persuasive strategies to functional elements of an intervention [the “theory-intervention gap”; (35)]. Previous studies mainly focused on which persuasive strategies are likely effective to increase PA, but these findings do not offer a guideline on how to operationalize the strategy. For example, the literature shows that the timing of reminders is important in PA interventions to ensure that people redirect their focus to the goal-directed behavior [performing exercise; (33)]. However, its specific interpretation, thus, what exactly are preferred or suitable time points to receive a reminder for exercise, remains unknown. To make this translation, it is important to involve end users of the intervention, in this case an exercise app. It is known that digital applications that are well fit to the user are used more often and longer (36), thereby increasing the chance to contribute to long-term health behavior change and in the end to a healthier society. By collecting input on needs and wishes of end users, the current study provides an important step in bridging the gap between the theoretically embedded persuasive strategies and their practical operationalization.

Interestingly, the findings concerning the needs and wishes of the participants of this study were not always aligned with the behavioral theories. For instance, in our study, the end-users indicated to prefer goals that match their abilities and that they perceive as (relatively) easy to achieve. This type of goals was appreciated, as this provides regular success experiences and thereby boost participants' motivation to continue regular PA. This is partly in contrast with the literature, which argues that realistic but challenging goals leads to better performance, rather than realistic but easy-to-achieve goals or goals that match their current abilities (37). Cases such as these, in which user preferences are not in accordance with existing theories, can provide a challenge for developers of exercise interventions. Further research is necessary to explore the discrepancies between what people want and what is effective according to existing theories and how to align these different views in the design and operationalization of persuasive strategies in apps. Concerning goal setting, a balance between both might be found in combining them. Thus, an app could be designed with an option to set ability-appropriate goals toward more distant and challenging goals. This way, participants experience regular success, and an exercise environment that supports persistence is designed.

Furthermore, the results of our focus group are also occasionally inconsistent with earlier focus group studies on exercise apps. For instance, where previous research indicates that active runners and cyclists prefer to monitor, for example, speed and distance traveled for performance reasons (38), this is different for our participants who would prefer to track calories burned and exercise time. This is probably due to our target group, which is more focused on improving health than on improving performance.

It is particularly noteworthy that participants' main goal to use an exercise app was to increase or maintain a healthy lifestyle. Participants explained that all the persuasive strategies should be designed and implemented in a way that fosters this motivation. For instance, they wish to monitor their progress toward their health-related goals and prefer feedback and reminders on, for example, the amount of time they still have walk that day to stay healthy. From a theoretical standpoint, this is also an interesting start point for future PA interventions. Namely, the self-determination theory (SDT) explains that such future intrinsic PA goals have a beneficial effect on effort expenditure, performance, autonomous exercise motivation, long-term persistence, and even sport club membership (39).

### Strengths and Limitations

Due to the specific target group of the current study, the results have limited translatability to other groups that have been found to not reach the recommended amount of PA in daily life, such as elderly and youth (5). Additional research is needed to explore the best operationalization for different target groups. In addition, living in different environments and being part of different cultures might result in different preferences regarding apps for outdoor exercise. In this perspective, a next step in the PAUL project that is currently performed is a similar focus group study in São Paulo (Br). This study will provide more insight into the differences in preferences for PA applications in different environments and cultures.

### Implications for Practice and Future Research

Concerning future directions for mHealth developments, findings on preferences for rewards, praise and encouragement further stress the need to adapt app features to the needs of specific target groups. Where most commercial apps use rewarding systems and apply competition in the configuration of these reward systems, this does not fit the target group of the inactive urban resident. In contrast to more physically skillful people who seek competition and are not reluctant against social comparison (40), our target group tends to dislike competition in exercise. The potential app users argued that they do not perceive themselves as very skillful in sports and that it is no fun to always come in last. In line with their preferences, it would be beneficial for this group of novice exercisers if exercise apps would reward them on personal growth rather than comparison with others. Moreover, their PA could benefit from other forms of social influence such as encouragement and compliments. Encouragement, for example, from friends and family, has consistently been related to an increase in physical activity (41, 42). Theories on motivation such as self-determination theory [SDT; (43, 44)] argue that competitive environments can have a detrimental effect on (sports) participation [e.g., (45, 46)]. Since you have no control over your competitors, perceived competence is quickly shattered by a defeat, and consequently, motivation and participation decline. When the rewarding system would be designed with a focus on personal growth instead, this leads to an increase in a person's feeling of competence and consequently leads to an increase in intrinsic motivation and adherence to the exercise program (45). We propose developers of exercise apps for novice exercisers to reward their users on the basis of personal growth and development instead of competition.

Another challenge for developers of future exercise interventions lies in the finding that preferences regarding the design characteristics of persuasive strategies were considerably divided between people, especially with regard to feedback and reminders. For instance, whether people wish to receive feedback before, during, or after exercise (the timing of feedback) varied considerably. Participants only wished to receive reminders for exercise in a, for them, relevant moment, which varied from only in the morning or evening to only during work days or weekends and a variety of specific time points during the day (e.g., only between 12:00 and 13:00). Current findings thus illustrate the need for an app that is adjusted to the more general target group that the user belongs to (e.g., inactive, focused on improving health, urban resident) as well as tailored to the individual user. A variety of studies found personalization of feedback to be beneficial for maximizing success of health interventions (47). A strategy that shows promising results to realize personalization of health apps is reinforcement learning (RL). With RL, adaptive interventions can be provided in which peoples' individual and fluctuating needs can be addressed (47, 48). However, although available studies show promising results (49), studies that apply RL to health interventions are scarce. Therefore, future research that explores the opportunities of reinforcement learning for mHealth interventions is needed.

### Conclusions

In conclusion, the current study provides a first step in the translation of theoretically embedded persuasive strategies into functional elements of an exercise app. Thereby, we provide guidance for developers of exercise interventions for designing mHealth applications to increase PA in inactive adults in urban environments. Findings show a need for an app that is adjusted to the target group and can be adapted on an individual level. Inactive urban residents in general preferred functionalities (e.g., goal setting, feedback, and monitoring) related to health guidelines and a rewarding system that is based on personal growth rather than competition. Moreover, especially with regard to feedback and reminders, there was the need for a personalized app. People aim to receive feedback and reminders in a, for them, relevant moment, which illustrates that optimizing timing, for example, via reinforcement learning is a promising strategy. Future research should further explore the possibilities of reinforcement learning for personalization of exercise apps. Moreover, future research should be directed at handling the discrepancy that sometimes exist between peoples' wishes and directions from existing theories with regard to the specific implementation of persuasive strategies. Finally, as the current study gives insight on app characteristics for a specific target group (Dutch healthy inactive urban residents), future studies should explore the practical implementation of persuasive strategies for different target groups and different cultures.

## Data Availability Statement

The datasets generated for this study are available on request to the corresponding author.

## Ethics Statement

The studies involving human participants were reviewed and approved by ethics review board of the Utrecht University. The patients/participants provided their written informed consent to participate in this study.

## Author Contributions

NN and MS performed the focus groups. NN performed the data analysis and wrote the first draft of the manuscript. All authors contributed to the conception and design of the study, manuscript revision, read, and approved the submitted version.

## Conflict of Interest

The authors declare that the research was conducted in the absence of any commercial or financial relationships that could be construed as a potential conflict of interest.

## Footnotes

^1^KC, Koen Castelein (master student in Human Movement Sciences, VU University, Amsterdam).
